# Supplementary material for: Development of a Systematic qPCR Array for Screening GM Soybeans
Source: Foods. 2021 Mar 13;10(3):610. doi: 10.3390/foods10030610 (PMC8001275; doi:10.3390/foods10030610)
Supplement: Supplementary file 1 [file foods-10-00610-s001.pdf]

Supplementary data 1. The results of monitoring test using universal screening method

Product type	Sample no.	Results			Events expected to be mixed in samples
		lectin	P-35S	T-nos	
Soybean Powder	1	+	-	-	MON87701, MON87705, MON87708, MON87751, MON87769, MON89788, DP305423-1, DP356043-5, CV127, DAS-44406-6, DAS-68416-4, DAS-81419-2
	2	+	-	-	
	3	+	-	-	
Dried Cereal	4	+	-	-	
	5	+	-	-	
	6	+	-	-	
	7	+	-	-	
	8	+	-	-	
	9	+	-	-	
	10	+	-	-	
	11	+	-	-	
Soy Milk	12	+	+	+	RRS, FG72, A2704-12, A5547-127, SYHT0H2<br>MON87701, MON87705, MON87708, MON87751, MON87769, MON89788, DP305423-1, DP356043-5, CV127, DAS-44406-6, DAS-68416-4, DAS-81419-2
Tofu	13	+	+	+	RRS, FG72, A2704-12, A5547-127, SYHT0H2<br>MON87701, MON87705, MON87708, MON87751, MON87769, MON89788, DP305423-1, DP356043-5, CV127, DAS-44406-6, DAS-68416-4, DAS-81419-2
	14	+	-	-	MON87701, MON87705, MON87708, MON87751, MON87769, MON89788, DP305423-1, DP356043-5, CV127, DAS-44406-6, DAS-68416-4, DAS-81419-2
	15	+	+	+	RRS, FG72, A2704-12, A5547-127, SYHT0H2<br>MON87701, MON87705, MON87708, MON87751, MON87769, MON89788, DP305423-1, DP356043-5, CV127, DAS-44406-6, DAS-68416-4, DAS-81419-2
	16	+	+	+	RRS, FG72, A2704-12, A5547-127, SYHT0H2<br>MON87701, MON87705, MON87708, MON87751, MON87769, MON89788, DP305423-1, DP356043-5, CV127, DAS-44406-6, DAS-68416-4, DAS-81419-2
	17	+	+	+	RRS, FG72, A2704-12, A5547-127, SYHT0H2<br>MON87701, MON87705, MON87708, MON87751, MON87769, MON89788, DP305423-1, DP356043-5, CV127, DAS-44406-6, DAS-68416-4, DAS-81419-2

	18	+	+	+	RRS, FG72, A2704-12, A5547-127, SYHT0H2 MON87701, MON87705, MON87708, MON87751, MON87769, MON89788, DP305423-1, DP356043-5, CV127, DAS-44406-6, DAS-68416-4, DAS-81419-2
	19	+	-	+	RRS, FG72, MON87701, MON87705, MON87708, MON87751, MON87769, MON89788, DP305423-1, DP356043-5, CV127, DAS-44406-6, DAS-68416-4, DAS-81419-2
	20	+	+	+	RRS, FG72, A2704-12, A5547-127, SYHT0H2 MON87701, MON87705, MON87708, MON87751, MON87769, MON89788, DP305423-1, DP356043-5, CV127, DAS-44406-6, DAS-68416-4, DAS-81419-2
	21	+	-	+	RRS, FG72, MON87701, MON87705, MON87708, MON87751, MON87769, MON89788, DP305423-1, DP356043-5, CV127, DAS-44406-6, DAS-68416-4, DAS-81419-2
	22	+	-	+	RRS, FG72, MON87701, MON87705, MON87708, MON87751, MON87769, MON89788, DP305423-1, DP356043-5, CV127, DAS-44406-6, DAS-68416-4, DAS-81419-2
Snack	23	+	-	-	MON87701, MON87705, MON87708, MON87751, MON87769, MON89788, DP305423-1, DP356043-5, CV127, DAS-44406-6, DAS-68416-4, DAS-81419-2

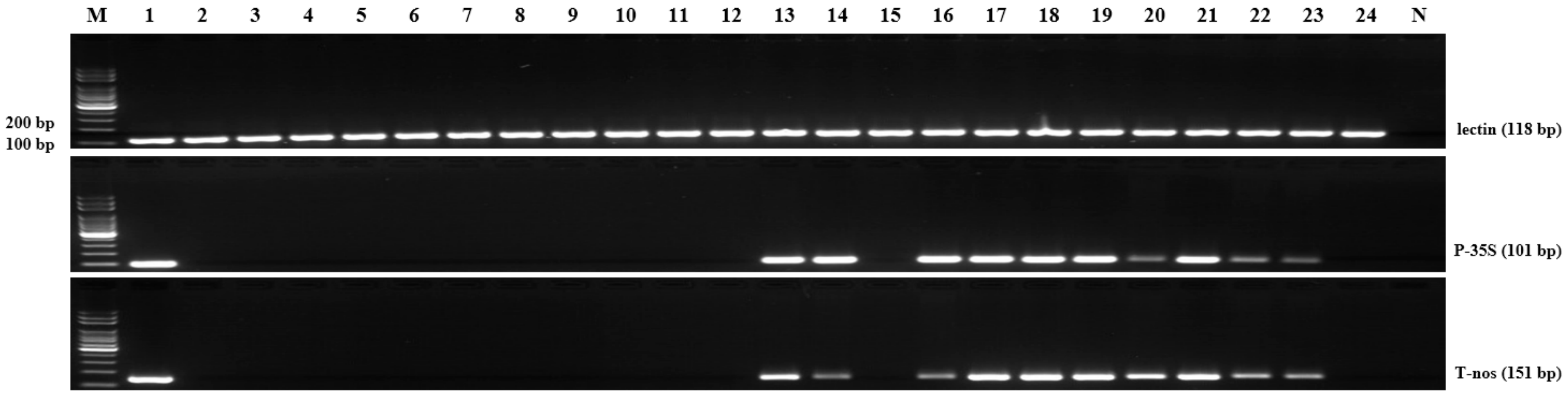

Lane M, 100 bp DNA Ladder; lane 1, Positive control; lanes 2-24, processed foods samples 1-23; lane N, no template
